# Data on megakaryocytes in the bone marrow of mice exposed to formaldehyde

**DOI:** 10.1016/j.dib.2015.12.058

**Published:** 2016-02-05

**Authors:** Yuchao Zhang, Cliona M. McHale, Xudong Liu, Xu Yang, Shumao Ding, Luoping Zhang

**Affiliations:** a Section of Environmental Biomedicine, Hubei Key Laboratory of Genetic Regulation and Integrative Biology, College of Life Science, Central China Normal University, Wuhan, China; b Division of Environmental Health Sciences, School of Public Health, University of California, Berkeley, CA, United States

## Abstract

Previously, we reported that occupational exposure to formaldehyde (FA) exposure in factory workers reduced platelet counts, *http://dx.doi.org/10.1158/1055*-*9965.EPI*-*09*-*0762*[1], while exposure in mice increased platelet counts *http://dx.doi.org/10.1371/journal.pone.0074974*[2]. Bone marrow megakaryocyte (MK) numbers were also increased in exposed mice, as determined qualitatively. The data presented here are from a quantitative evaluation of MK numbers in the bone marrow histopathological slides from the previous FA exposure experiments in mice. Bone marrow slides were prepared using a single 5 μm section of femur from 2 mice randomly selected from each exposure group (*n*=9) treated with 0, 0.5 and 3.0 mg/m^3^ FA by nose-only inhalation. MKs were systemically counted and average MK frequency was calculated as the total MK per slide divided by the number of fields evaluated. Data are presented visually as microscopy views and graphically as MK frequency.

## **Specifications Table**

**Table:** 

Subject area	Biology
More specific subject area	Occupational and environmental toxicology
Type of data	Figures
How data was acquired	Microscope
Data format	Raw (Fig. 1) and analyzed (Fig. 2).
Experimental factors	Male Balb/c mice were exposed to 0, 0.5 and 3.0 mg/m^3^ FA (*n*=9 per group) by nose-only inhalation for two weeks to mimic occupational exposure. Bone marrow histology slides were prepared from 2 mice randomly selected from each exposure group and MKs were counted.
Experimental features	MKs were systematically counted in each scorable field on each slide under 20× magnification and MK frequency was calculated as the total MK per slide divided by the number of fields evaluated.
Data source location	Wuhan, China and Berkeley, California, USA.
Data accessibility	Data is in this article.

## **Value of the data**

• Quantitatively confirms that MK numbers are increased in the bone marrow of FA-exposed mice in a dose-dependent manner.
• Strengthens evidence that FA induces bone marrow toxicity, particularly in myeloid progenitor cells.
• May stimulate research on the underlying mechanisms of FA-induced myeloid toxicity and differences in response between mouse and human.

## 1. Data

**Fig. 1:**
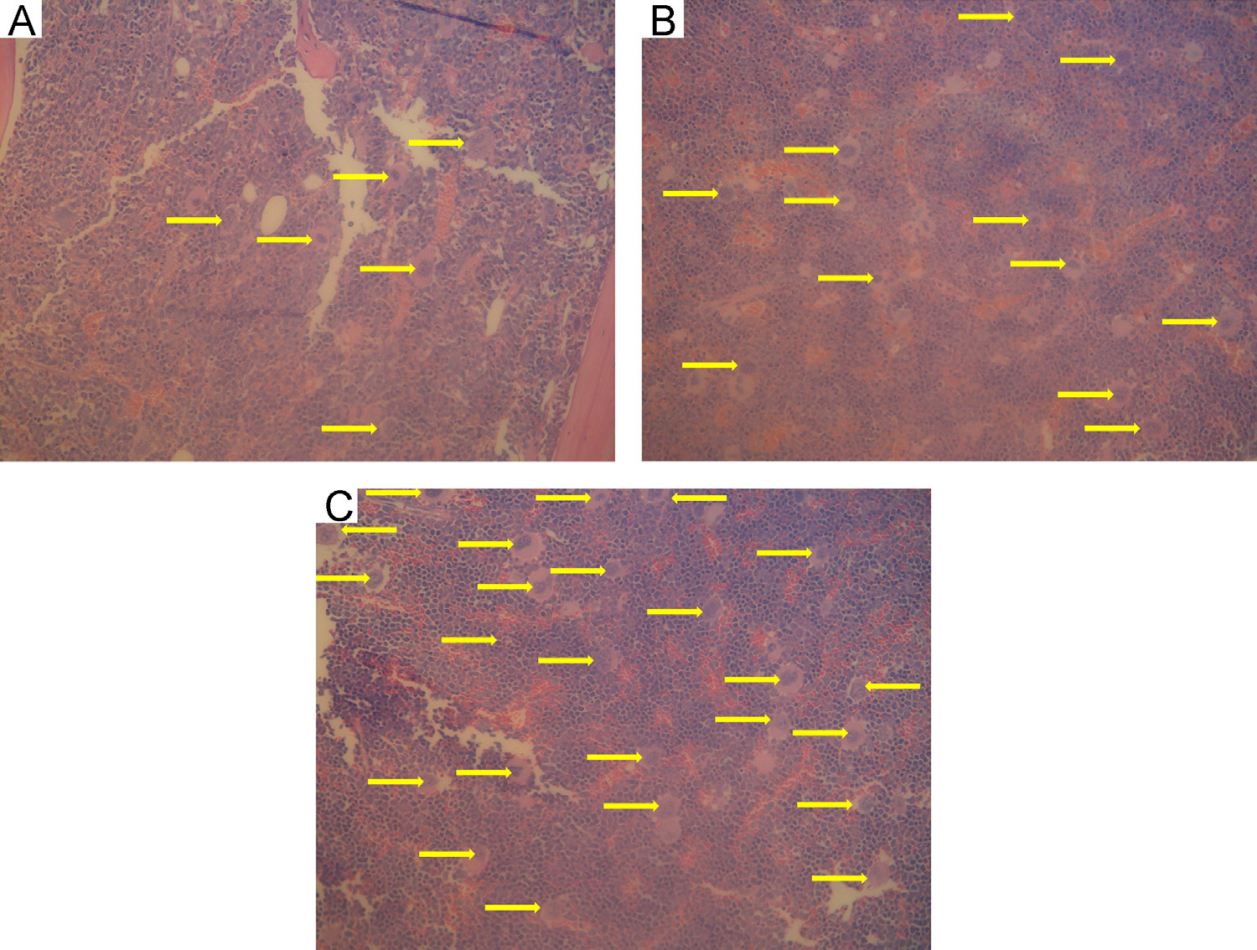
Representative microscopy fields showing the numbers of MKs observed at (a) 0, (b) 0.5, and (c) 3.0 mg/m^3^ FA. MKs are indicated by arrows. 20× magnification.

**Fig. 2:**
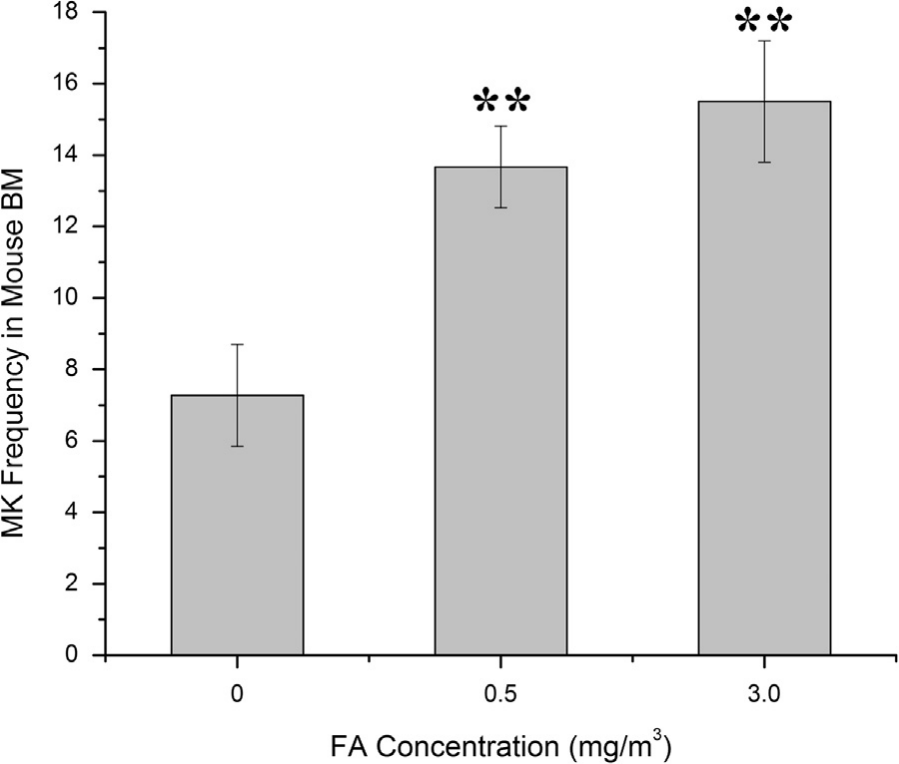
MK frequency in bone marrow of FA-exposed and unexposed control mice. MK frequency (the total number of MKs divided by the total number of scorable fields) under 20× magnification is shown for 0, 0.5 and 3.0 mg/m^3^ FA in a single bone marrow section from triplicate experiments. ^**^p<0.01.

Previously, we reported that occupational formaldehyde (FA) exposure decreased counts of circulating mature blood cells, including platelets [1]. However, in mice exposed to FA, we found an increase in the numbers of platelets circulating in blood [2]. Further, there was an apparent concomitant increase in the number of bone marrow megakaryocytes (MK), the precursors of platelets [3], [4], as determined qualitatively from histopathological bone marrow slides [2]. Here, a quantitative re-evaluation of the bone marrow slides was conducted by systematically counting the MK cells using a microscope. Data on MKs from FA-exposed and control mice are presented qualitatively (representative microscopy fields, Fig. 1) and quantitatively (MK frequencies, Fig. 2).

Visual examination of MK cells on the slides revealed increased numbers with increasing FA dose (Fig. 1).

Quantitatively, the total number of MKs scored (80, 82, and 186) increased respectively with increasing FA doses (unexposed controls, 0.5 mg/m^3^ and 3.0 mg/m^3^). The corresponding numbers of scorable fields were 11, 6 and 12, respectively. As shown in Fig. 2, the resulting MK frequency (the total number of MKs divided by the total number of scorable fields) was significantly increased in the FA-exposed mice at 0.5 mg/m^3^ (13.67, *p*<0.01) and 3.0 mg/m^3^ (15.50, *p*<0.01) compared with the unexposed control group (7.27) (Fig. 2) and a dose-dependent effect was apparent (*p*_trend_=0.001).

The MK frequency data quantitatively shows that MK numbers are increased in FA-exposed mice compared with controls (Fig. 2).

## 2 Experimental design, materials and methods

### 2.1 Experimental design

Male Balb/c mice were exposed to 0, 0.5 and 3.0 mg/m^3^ FA (*n*=9 per group) by nose-only inhalation for two weeks to mimic occupational exposure (exposure for 5 days followed by no exposure for two days). Bone marrow histology slides were prepared from 2 mice that were randomly selected from each exposure group and MKs were systematically counted using a microscope under 20× magnification.

### 2.2 Experimental animals and FA exposure

All experimental procedures were approved by the Office of Scientific Research Management of Huazhong Normal University (Huazhong, China) through certification of Application for the Use of Animals, dated November 8, 2011 (approval ID: CCNU-SKY-2011-008). The experimental protocol was detailed previously [2]. In brief, male Balb/c mice (5–6 wks old, 22±1.5 g) were purchased (Hubei Province Experimental Animal Center, Wuhan, China) and housed in standard environmental conditions (12 h light–dark cycle, 50–70% humidity and 20–25 °C) in a state-certified animal facility. The mice were quarantined for at least 7 days and then divided randomly into three exposure groups (9 mice in each group): 0, 0.5, and 3.0 mg/m^3^, reflecting current and historical Chinese occupational exposure limits. Mice were exposed to FA vapor made from 10% formalin via nose-only inhalation for 5 consecutive days per week (8 h a day, 9 am–5 pm), for two weeks. FA concentrations were monitored every 2 h using a Gaseous FA Analyzer (4160i2, Interscan, Simi Valley, CA, USA). Two mice from each group were randomly selected for bone marrow histopathological studies.

### 2.3 Bone marrow histology protocol

A single mouse femur was removed from each mouse. Bone marrow sections were prepared by Biossci Biotechnologies Company Limited, Hubei, using the company’s standard procedure. Briefly, femurs were fixed in Bouin solution containing saturated 2,4,6-trinitrophenol: formalin: glacial acetic acid (15:5:1 v/v/v) at room temperature for 24 h. Femurs were transferred to 70% ethanol and subjected to decalcification in 10% EDTA for 1 week. The bone marrow tissues then underwent paraffin processing and were embedded in paraffin. For each mouse and dose, a single longitudinal 5 μm section was prepared using a microtome, mounted on a slide, stained with hematoxylin and eosin (H&E) and covered with a cover slip. Stained sections were examined qualitatively and quantitatively by two experienced pathologists in a double-blinded fashion under a microscope (Leica DM 4000B, Berlin, Germany).

### 2.4 Counting of megakaryocytes

MKs are 10–15 times larger than a typical red blood cell, averaging 50–100 μm in diameter, and have large lobulated nuclei. Thus, MKs are easy to visually identify and count by microscopy. Trained researchers viewed each entire slide under 10× magnification to identify unscorable areas with scratches or missing material, and then counted the MKs in all scorable fields under 20× magnification. The observers systematically scored the entire slide by manually recording each field (X- and Y-stage) and the number of MKs therein. Under 20× magnification, each field was calculated to be approximately 3.236×10^−9^ m^2^ (or 3.236×10^3^ µm^2^). For each slide, we calculated the MK frequency as the average number of MKs per field (the total number of MKs scored divided by the total number of fields assessed).

### 2.5 Statistical analysis

One-way analysis of variance (ANOVA) combined with Fisher’s Protected Least Significant Difference (PLSD) *t*-test was used to determine significant differences between exposure groups and dose-response was analyzed by trend test. *P* values and *P*_trend_ values <0.05 were considered significant. Data analyzes were carried out using SPSS ver13 (SPSS, Chicago, IL, USA). Statistical graphs were generated using Origin 8.0 software (OriginLab, Berkeley, CA, USA). Data were measured in triplicate and are presented as mean and standard error of the mean.
